# The impact of integrative medicine on quality of life in patients with diabetes mellitus and cancer

**DOI:** 10.1007/s00520-026-10511-6

**Published:** 2026-03-17

**Authors:** Sameer Kassem, Orit Gressel, Noah Samuels, Nili Stein, Benjamin Glaser, Eran Ben-Arye

**Affiliations:** 1 Rappaport Faculty of Medicine, Technion-Israel Institute of Technology, Haifa, Israel; 2 Department of Internal Medicine, Carmel Medical Center, Clalit Health Services, Haifa, Israel; 3 Integrative Oncology Program, The Oncology Service, Zebulun Medical Centers, Clalit Health Services, Carmel &, LinHaifa, Israel; 4 Center for Integrative Complementary Medicine, Faculty of Medicine, Shaare Zedek Medical Center, Hebrew University of Jerusalem, Jerusalem, Israel; 5 Department of Community Medicine and Epidemiology, Carmel Medical Center, Haifa, Israel; 6 Department of Endocrinology and Metabolism, Faculty of Medicine, Hadassah Medical Center, Hebrew University of Jerusalem, Jerusalem, Israel

**Keywords:** Integrative complementary medicine, Diabetes, Quality of life, Supportive care, Integrative medicine

## Abstract

**Purpose:**

Integrative complementary medicine (ICM) combines complementary therapies with conventional supportive and palliative care to address quality of life (QoL). Diabetes mellitus (DM) is prevalent among patients with cancer and has been associated with worse health-related QoL. We examined the impact of an ICM program on QoL-related concerns among patients being treated for cancer, with vs. without DM.

**Methods:**

This prospective, controlled and pragmatic study examined a 6-week ICM program, comparing DM to non-DM patients using the ESAS (Edmonton Symptom Assessment Scale) and EORTC QLQ-C30 (European Organization for Research and Treatment of Cancer Quality of Life Questionnaire) tools. The threshold for statistical significance was established at a *P*-value of less than 0.05.

**Results:**

Of 671 patients, 135 (20%) had DM, with similar baseline QoL-related concerns (pain, fatigue, gastrointestinal and emotional concerns) in both groups. DM patients (vs. non-DM) responded significantly less on drowsiness on ESAS (*P* = 0.047); and emotional functioning on EORTC (*P* = 0.017). Compared to baseline, the non-DM group showed significant improvement in fatigue and sleep quality (on ESAS and EORTC; *P* < 0.001), while the DM group did not show any improvement for these concerns.

**Conclusion:**

Patients with cancer and DM showed a less significant response to ICM treatments when compared to non-DM patients, emphasizing the need for identifying this sub-population of patients while creating specialized ICM programs tailored to their needs. Future research should focus on exploring the impact of ICM programs on patients with both DM and cancer.

Trial registration

ClinicalTrials.gov NCT01860365, 2013-05-21.

## Introduction

Integrative complementary medicine (ICM) combines complementary therapies with conventional medical treatments to address overall well-being, focusing on the physical, emotional, mental, and spiritual aspects of health. Acupuncture, touch, and mind-body therapies are becoming increasingly recognized as safe and effective supplements to conventional medicine for quality of life (QoL)-related concerns among patients with chronic illness, including diabetes mellitus (DM) [1]. Nearly half (48%) of patients diagnosed with DM report using complementary medicine (herbs, yoga, relaxation, acupuncture, ayuverda, biofeedback, Reiki therapy, hypnosis, massage, naturopathy, and homeopathy), in conjunction with their conventional DM treatment [2]. Co-morbidity with DM and cancer is common and has been associated with a worse prognosis [3] and poorer health-related QoL outcomes [4]. The bi-directional relationship between DM and cancer can be seen by an increased risk for developing several cancers in diabetic patients, independent of shared risk factors such as obesity and age. Conversely, certain cancers and cancer therapies (e.g., pancreatic cancer, immunotherapies, glucocorticoids, and some targeted agents) can precipitate DM, either through direct effects on pancreatic beta cells or by inducing insulin resistance.

ICM programs can be found in leading oncology centers in the USA and across the globe [5]. A large body of evidence supports the safe and beneficial effects of ICM modalities. The American Society of Clinical Oncology (ASCO) and Society for Integrative Oncology (SIO) collaboratively published clinical practice guidelines endorsing the use of ICM for patients with cancer. To date, the SIO-ASCO guidelines have addressed the role of ICM in the treatment of female patients with breast cancer [6, 7], as well as in managing cancer-related symptoms in both genders, including cancer-related pain [8]; cancer-related fatigue [9]; and anxiety and depression [10]. Real-world pragmatic research has shown that high adherence to a 6–12-week ICM intervention is associated with improved QoL-related outcomes such as pain and emotional functioning in patients with breast, gynecological [11], and lung cancer [12].

Despite this relationship between cancer and DM, little research has been published on the impact of ICM on QoL-related outcomes in patients with both conditions. This prospective, controlled, and pragmatic study explored the impact of a 6-week, patient-tailored ICM treatment program on patients with cancer undergoing active oncology treatment, comparing those with both cancer and DM to those without DM.

## Research design and methods

The study was prospective, controlled, and pragmatic, and took place at the Lin, Zebulon, and Carmel medical centers in northern Israel (from May to October 2023). Eligible participants were patients with a diagnosis of cancer; 18 years of age or older; and undergoing chemotherapy, biological treatment, and/or palliative care for solid tumors. The study protocol was approved by the Institutional Review Board at the Carmel Medical Center in Haifa, Israel, and registered at ClinicalTrials.gov (NCT01860365).

### Integrative oncology program

Oncology healthcare providers, including oncologists, nurse oncologists, and psycho-oncologists, referred patients at the study centers to an integrative physician (a medical doctor trained in ICM) consultation. These healthcare providers were required to specify at least one QoL-related indication for the referral (e.g., pain, anxiety, or fatigue). During the consultation (lasting approximately an hour), the integrative physician and patient explored QoL-related concerns and well-being; co-defining treatment goals; and then scheduling a 6-week ICM treatment program. The ICM program consisted of 6 weekly patient-tailored sessions, each lasting between 30 and 45 min. The study included only those patients who participated in at least 4 treatment sessions during the 6-week period following consultation.

Complementary medicine therapies provided during these sessions included at least one of the following: acupuncture; manual therapies (e.g., acupressure, reflexology, manual Anthroposophical techniques, techniques that often involve gentle, hands-on approaches such as massage, tactile stimulation, and movement exercises designed to support physical, emotional, and spiritual well-being); movement therapies (e.g., Qi Gong); guidance on the effective and safe use of herbal and dietary supplements; and mind-body-spirit therapies (e.g., guided imagery). Standard supportive/palliative and psycho-oncology care was provided throughout, including consultations and medications as recommended by oncology and palliative health care providers.

### Study outcomes

QoL-related outcomes were scored at baseline (initial ICM consultation) and at 6 weeks. The Edmonton Symptom Assessment Scale (ESAS) was used to assess the severity of patient QoL-related concern over the previous 24-h [13]; and the European Organization for Research and Treatment of Cancer Quality of Life Questionnaire (EORTC QLQ-C30) for the previous week [14]. The 2 questionnaires were administered to all participants in both study groups (DM vs. non-DM).

### Statistical analysis

It was determined that at least 50 patients would be needed in each study group (DM vs. non-DM) to assess the impact of the ICM program on study outcomes (OpenEpi program, Microsoft). This would allow for an alpha error of 0.05 and beta error of 0.2 (power 80%), for a difference of 25% on ESAS scores. Statistical analyses were conducted using SPSS Statistical software. Continuous variables are presented using means and standard deviations (SDs) or medians and inter-quartile range (IQR). Categorical variables are presented using numbers and proportions. Comparisons between baseline characteristics of the two study groups were performed using a Chi square test for categorical variables and an independent t-test or Mann–Whitney for continuous variables. Differences in QoL-related concerns between baseline and 6-week follow-up visits were analyzed separately for each study group using Wilcoxon sign rank test. Changes in QoL-related concern scores between the two groups were compared using a Mann–Whitney test.

## Results

Of 671 oncology patients found to be highly adherent to the ICM program at the study centers, 135 (20%) had a comorbid diagnosis of DM. Table 1 compares baseline demographic and clinical characteristics of the DM vs. non-DM groups, including cancer-related parameters; prior/current use of complementary medicine practices; health beliefs and expectations; and comorbidities (hypertension, etc.). Patients with DM were more likely to be older, with a higher proportion of males. They also had significantly more baseline comorbidities, with a greater Charlson Comorbidity Index score; were more likely to present with metastatic disease; and had a higher prevalence of gastrointestinal cancer (31.9% vs. 16.8%) and a lower prevalence of breast cancer (32.6% vs. 56.2%) as their primary tumor. At the same time, patients in both groups reported similar baseline QoL-related concerns during the initial consultation, most commonly fatigue and gastrointestinal symptoms.

**Table 1:** Baseline characteristics of patients undergoing oncology treatment with diabetes versus non-diabetes

Characteristic	Diabetes *n* = 135	Non-diabetes *n* = 536	*P*-value
**Age** Mean ± SD	67.1 ± 8.7	57.8 + 13.2	< 0.001
**Gender/sex** Female	91 (67.4)	455 (84.9)	< 0.001
**Primary language** Hebrew	98 (72.6)	368 (68.7)	0.375
**Country of birth** Israeli born	75 (55.6)	335 (62.5)	0.139
**Residence** Haifa vs. suburbs & periphery	97 (71.9)	361 (67.4)	0.315
**Social economic status** Low Middle High	41 (30.8) 68 (51.1) 24 (18)	171 (32.3) 243 (45.9) 115 (21.7)	0.503
**Primary cancer site:** Breast Gynecological cancers Gastrointestinal Lung Prostate Urinary Other	44 (32.6) 22 (16.3) 43 (31.9) 15 (11.1) 2 (1.5) 7 (5.2) 2 (1.5)	301 (56.2) 87 (16.2) 90 (16.8) 41 (7.6) 5 (0.9) 5 (0.9) 4 (0.7)	< 0.001 0.985 < 0.001 0.194 0.633 0.004 0.348
**Cancer recurrence** Yes	29 (21.5)	98 (18.3)	0.402
**Cancer metastasis** Yes	59 (45)	174 (33.4)	0.013
**Oncology treatment setting** Adjuvant Neoadjuvant Palliative	33 (25.8) 39 (30.5) 56 (43.8)	175 (34.2) 177 (34.6) 159 (31.1)	0.022
**Prior CM use** (non-cancer related) Yes	87 (64.4)	358 (67.0)	0.568
**Cancer-related CM use:** Yes	50 (37.3)	242 (45.4)	0.092
Major QoL-related concerns** at the IP visit: Pain Emotional concerns Gastro-intestinal concerns Fatigue	82 (62.1) 89 (67.4) 110 (83.3) 105 (79.5)	324 (61.8) 365 (69.9) 395 (75.5) 430 (82.2)	0.951 0.578 0.056 0.478
**Comorbidities:** Hypertension Hyperlipidemia Obesity Ischemic heart disease Chronic renal failure Cerebrovascular accident **Charlson Comorbidity Index**	105 (77.8) 124 (91.9) 74 (54.8) 25 (18.5) 20 (14.8) 12 (8.9) 6.1 ± 2.0 6 (5; 7)	149 (27.8) 294 (54.9) 103 (19.2) 29 (5.4) 16 (3.0) 9 (1.7) 3.3 ± 1.8 3 (2; 5)	< 0.001 < 0.001 < 0.001 < 0.001 < 0.001 < 0.001 < 0.001

The percentage does not up to 100% because of missing information in some categories

*CM* complementary medicine, *IP* Integrative physician, *QoL* quality of life

^*^Data based on a Likert scale scored from 1 (very slightly agree) to 7 (agree very much)

^**^Qol-related concerns as determined during the integrative physician consultation

The ICM modalities provided to patients from both groups in the cohort are presented in Table 2. No significant differences were found between the groups vis-à-vis the ICM modalities used in their treatment, with acupuncture the most frequently used modality (> 95% of patients in both groups), followed by manual-movement therapies.

**Table 2:** Integrative complementary medicine (ICM) modalities used to treat patients with cancer and diabetes (vs. non-diabetes) during the 6-week following ICM consultation

ICM modality	Diabetes *n* = 135	Non-diabetes *n* = 536	*P*-value
Acupuncture	*N* = 124 121 (97.6)	*N* = 487 463 (95.1)	0.225
Manual-movement	*N* = 124 108 (87.1)	*N* = 487 399 (81.9)	0.172
Herbal medicine	*N* = 124 88 (71.0)	*N* = 487 363 (74.3)	0.448
Mind-body	*N* = 122 62 (50.8)	*N* = 278 278 (57.2)	0.204
Anthroposophic treatment	*N* = 124 32 (25.8)	*N* = 487 132 (27.1)	0.771
Number of IO treatment days Mean ± SD	*N* = 127 7.3 ± 6.4	*N* = 493 6.9 ± 1.9	0.858
Number of IO interventions Mean ± SD	*N* = 127 19.49 ± 8.2	*N* = 493 18.9 ± 7.9	0.499

**Fig. 1:**
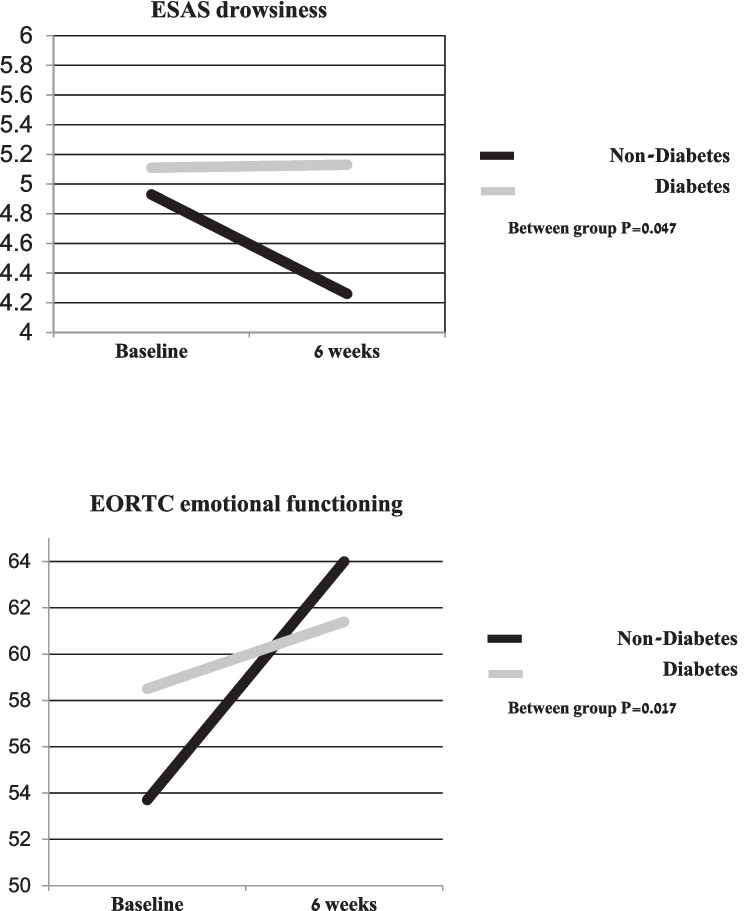
Changes in ESAS drowsiness and EORTC emotional functioning after 6 weeks of integrative complementary medicine (ICM) intervention comparing diabetes group to non-diabetes groups

Table 3 presents ESAS and EORTC scores, from the initial consultation to the end of the 6-week ICM intervention. No differences in QoL-related scores between the two groups were found at baseline, except for appetite and well-being on EORTC (over the past week), which were significantly worse in the DM group. While the DM group showed improvement in only some of the QoL domains, the non-DM group exhibited a favorable response for all QoL-related concerns after 6 weeks of ICM treatments. Between-group comparison showed that patients in the DM group reported significantly less improvement on drowsiness (ESAS, *P* = 0.047) and emotional functioning (EORTC, *P* = 0.017) scores (Fig. 1). Within-group improvement was found only in the non-DM group (not in the DM group) for fatigue, nausea, breathing and sleep (ESAS) and for fatigue and sleep (EORTC). Within-group improvement was shown in both groups for pain, depression, anxiety, appetite (ESAS), and well-being; and for pain, nausea and appetite (EORTC).

**Table 3:** Comparison of QoL-related concerns between initial and follow-up assessments after 6 weeks of integrative complementary medicine (ICM) intervention among patients in diabetes vs. non-diabetes groups

	**Diabetes** ***n* = 135**		**Non-diabetes** ***n* = 536**		
Mean score ± SD (median)	**Baseline**	**6 weeks**	**Baseline**	**6 weeks**	***P*-value**
**Edmonton Symptom Assessment Scale (ESAS)**					
Pain	4.67 ± 3.3 5(2; 8)	4.04 ± 3.40 4 (0; 7)	4.29 ± 3.02 5 (2; 7)	3.54 ± 3.01 3 (0; 6)	*P*^1=^0.206, *P*^2^ = 0.047 *P*^3^ < 0.001 *P*^4^ = 0.587
Fatigue	6.10 ± 2.89 7 (5; 8)	5.80 ± 3.08 6 (4; 8)	5.78 ± 2.67 6 (4; 8)	5.27 ± 2.83 5 (3;7)	*P*^1=^0.124, *P*^2^ = 0.315 *P*^3^ < 0.001, *P*^4^ = 0.382
Nausea	2.43 ± 3.09 1 (0; 5)	2.04 ± 2.89 0 (0; 3)	2.53 ± 3.04 1 (0; 5)	2.0 ± 2.68 0 (0; 4)	*P*^1=^0.593, *P*^2^ = 0.093 *P*^3^ < 0.001, *P*^4^ = 0.957
Depression	3.52 ± 3.41 3 (0; 6)	2.70 ± 3.11 1 (0; 5)	3.30 ± 3.16 3 (0;5)	2.46 ± 2.91 1 (0; 5)	*P*^1=^0.665, *P*^2^ < 0.001 *P*^3^ < 0.001, *P*^4^ = 0.947
Anxiety	3.70 ± 3.44 3 (0; 6)	2.92 ± 3.11 2 (0; 5)	3.94 ± 3.31 4 (1; 7)	2.85 ± 3.09 2 (0; 5)	*P*^1=^0.400, *P*^2^ = 0.013 *P*^3^ < 0.001, *P*^4^ = 0.315
Drowsiness	5.11 ± 3.08 5 (3; 8)	5.13 ± 3.0 5 (3; 7)	4.93 ± 3.04 5 (3; 7)	4.26 ± 3.15 4 (1; 7)	*P*^1=^0.479, *P*^2^ = 0.999 *P*^3^ < 0.001, *P*^4^ = 0.047
Breathing	2.21 ± 2.81 0 (0; 4)	1.79 ± 2.60 0 (0;3)	1.97 ± 2.74 0 (0; 4)	1.63 ± 2.57 0 (0; 3)	*P*^1=^0.351, *P*^2^ = 0.095 *P*^3^ = 0.004, *P*^4^ = 0.626
Appetite	4.97 ± 3.45 5 (2; 8)	4.26 ± 2.98 4 (2; 6)	4.23 ± 3.20 5 (1; 7)	3.66 ± 3.10 3 (1; 6)	*P*^1=^0.024, *P*^2^ = 0.036 *P*^3^ < 0.001, *P*^4^ = 0.915
Sleep	4.36 ± 3.24 5 (1; 7)	4.05 ± 2.99 4 (1; 6)	4.76 ± 3.3 5 (2; 7)	4.02 ± 2.99 4 (2; 6)	*P*^1=^0.199, *P*^2^ = 0.196 *P*^3^ < 0.001, *P*^4^ = 0.394
Well-being	5.88 ± 2.67 6 (4; 8)	5.36 ± 2.51 5 (4; 7)	5.36 ± 2.64 5 (4; 7)	4.78 ± 2.51 5(4;7)	*P*^1=^0.032, *P*^2^ = 0.029 *P*^3^ < 0.001, *P*^4^ = 0.798
**EORTC QLQ-C30 functional scales**					
Emotional functioning	58.5 ± 30.4 58 (42; 83)	61.4 ± 28.2 67 (42;83)	53.7 ± 30.3 58 (33;75)	64.0 ± 29.2 67 (42; 92)	*P*^1^ = 0.130, *P*^2^ = 0.268 *P*^3^ < 0.001, *P*^4^ = 0.017
**EORTC QLQ-C30 symptoms scales**					
Pain	53.6 ± 34.9 50 (33–83)	45.6 ± 37.3 42 (0; 79)	51.3 ± 34.4 50 (17; 83)	43.6 ± 34.1 33 (17; 67)	*P*^1^ = 0.500, *P*^2^ = 0.015 *P*^3^ < 0.001, *P*^4^ = 0.994
Fatigue	72.7 ± 27.0 78 (56; 100)	69.0 ± 25.6 67 (56; 89)	71.2 ± 27.8 78 (56; 100)	66.0 ± 27.3 67 (44; 89)	*P*^1^ = 0.666, *P*^2^ = 0.085 *P*^3^ < 0.001, *P*^4^ = 0.367
Nausea	21.1 ± 24.2 17 (0; 33)	12.8 ± 17.6 0 (0; 17)	21.1 ± 23.8 17 (0; 33)	16.1 ± 20.3 17 (0; 33)	*P*^1^ = 0.972, *P*^2^ < 0.001 *P*^3^ < 0.001, *P*^4^ = 0.381
Appetite	52.2 ± 39.3 67 (0; 100)	44.4 ± 35.7 33 (0; 67)	46.7 ± 38.1 33 (0; 67)	39.5 ± 37.4 33(0; 67)	*P*^1^ = 0.165, *P*^2^ = 0.018 *P*^3<^0.001, *P*^4^ = 0.990
Sleep	48.7 ± 38.3 33 (0; 83)	43.9 ± 37.3 33 (0; 67)	52.8 ± 37.5 67 (33; 100)	45.4 ± 36.2 33 (0; 67)	*P*^1^ = 0.305, *P*^2^ = 0.250 *P*^3^ < 0.001, *P*^4^ = 0.560

Functioning scale has scores ranging from 0 (greatest severity) to 100 (lowest severity), while symptom scales ranging from 0 (lowest severity) to 100 (greatest severity)

*ESAS* Edmonton Symptom Assessment Scale is a scale from 0 (lowest severity) to 10 (greatest severity), *EORTC QLQ-C30* European Organization for Research and Treatment of Cancer Quality of Life Questionnaire

^*^P-values are presented concerning the following comparisons between the study groups: *P*^1^ = compared DM and non-DM group scores at initial visit (baseline scores); *P*^2^ = within DM group score changes from initial to follow-up visit; *P*^3^ = within non-DM group score changes from initial to follow-up visit; *P*^4^ = between DM and non-DM group changes from initial to follow-up visit

## Discussion

The study findings suggest that, despite significant baseline differences between the two groups, a significant improvement was observed in both for QoL-related concerns identified during the initial consultation. The predominance of cancer-related concerns over those related to DM is to be expected, given the perception of cancer as an existential and life-threatening illness, in contrast to a chronic disease like DM. This heightened sense of danger may lead patients to prioritize concerns associated with cancer and its treatment, which tend to overshadow other health issues. While some concerns, such as fatigue and neuropathic pain, may be related to both the patient's cancer and DM, the dominance of cancer-related concerns most likely shapes their overall concerns and priorities during the integrative physician consultation.

An important finding of the study is that patients with both cancer and DM reported a significantly less improvement following the 6-week ICM intervention, particularly for drowsiness (ESAS) and emotional functioning (EORTC), when compared to patients without DM. These differences may be attributed to the increased burden of QoL-related concerns resulting from their DM and its complications, such as ischemic heart disease, cerebrovascular disease, and renal failure. Additionally, other baseline differences between the groups, such as age, may have contributed to the observed disparities in the response to ICM treatments.

The study cohort consisted exclusively of patients who demonstrated high adherence to the ICM program, ensuring that the observed responses were not influenced by gaps in attending the treatment sessions. Thus, the less significant improvement in response to ICM treatments observed in patients with both cancer and DM cannot be explained by their receiving less treatment, but rather an as-yet not understood form of "refractoriness" to these interventions. It is important to note that these findings are consistent with those of previous research indicating that patients with DM tend to experience poorer QoL-related outcomes and face a higher risk of adverse events during active oncology treatment [15, 16]. For example, patients with cancer and DM have been shown to suffer from more severe cancer-related fatigue, both before and after chemotherapy, with symptoms lasting up to 6 months post-chemotherapy among survivors of breast cancer [17]. This may, at least in part, be due to the additional burden of DM-related complications, comorbidities, or physiological factors that diminish the effectiveness of supportive interventions, highlighting the need for tailored approaches to improve QoL-related outcomes in this vulnerable patient population.

There are a number of study limitations which need to be addressed in future research, the most significant of which is the study's pragmatic and non-randomized methodology. While explanatory, randomized controlled trials (RCTs) may reduce the risk for a control selection bias, the pragmatic methodology used here enabled the assessment of two distinct groups (DM and non-DM), which were found to be significantly different in their baseline demographic, oncologic, and comorbidity-related characteristics. At the same time, both study groups reported largely similar QoL-related concerns at baseline, enabling a between-group comparison. Other limitations include the use of patient-reported outcomes, which allow for assessing only subjective, QoL-focused responses to the study intervention, as opposed to objective measures (e.g., relative dose intensity, which measures patient adherence to the planned chemotherapy regimen); and the limited generalizability of the study findings for other oncology centers in Israel and across the globe. Finally, the use of a multimodality ICM program, in which treatments were tailored to each patient, precludes reaching any conclusions regarding the effectiveness of individual ICM modalities such as acupuncture. Despite these limitations, the pragmatic patient-centered and patient-tailored approach of the study setting is more reflective of real-world ICM practice and can better examine the true impact of ICM models of care.

In conclusion, the present study found that despite similar baseline QoL-related concerns, the 6-week patient-tailored ICM treatment program described had a significantly greater impact on QoL-related concerns among patients with cancer who did not have a co-diagnosis of DM, compared to those with DM undergoing active oncology treatment. Further research is needed, within an explanatory RCT format and focusing on specific oncology patient populations, such as those with a comorbidity of DM.

## Data Availability

The datasets generated during and/or analyzed in the current study are available from the corresponding author upon reasonable request.
